# Sacubitril/valsartan in the treatment of systemic right ventricular failure

**DOI:** 10.1136/heartjnl-2020-318074

**Published:** 2021-01-15

**Authors:** Tjitske E Zandstra, Marieke Nederend, Monique R M Jongbloed, Philippine Kiès, Hubert W Vliegen, Berto J Bouma, Laurens F Tops, Martin J Schalij, Anastasia D Egorova

**Affiliations:** 1 CAHAL, Center for Congenital Heart Disease Amsterdam Leiden, Leiden University Medical Center, Leiden, The Netherlands; 2 Department of Anatomy & Embryology, Leiden University Medical Center, Leiden, The Netherlands; 3 CAHAL, Center for Congenital Heart Disease Amsterdam Leiden, Amsterdam University Medical Center, Amsterdam, The Netherlands; 4 Department of Cardiology, Leiden University Medical Center, Leiden, The Netherlands

**Keywords:** congenital heart disease, transposition of the great arteries, heart failure, complex congenital heart disease

## Abstract

**Objective:**

Pharmacological options for patients with a failing systemic right ventricle (RV) in the context of transposition of the great arteries (TGA) after atrial switch or congenitally corrected TGA (ccTGA) are not well defined. This study aims to investigate the feasibility and effects of sacubitril/valsartan treatment in a single-centre cohort of patients.

**Methods:**

Data on all consecutive adult patients (n=20, mean age 46 years, 50% women) with a failing systemic RV in a biventricular circulation treated with sacubitril/valsartan in our centre are reported. Patients with a systemic RV ejection fraction of ≤35% who were symptomatic despite treatment with β-blocker and ACE-inhibitor/angiotensin II receptor-blockers were started on sacubitril/valsartan. This cohort underwent structural follow-up including echocardiography, exercise testing, laboratory investigations and quality of life (QOL) assessment.

**Results:**

Six-month follow-up data were available in 18 out of 20 patients, including 12 (67%) patients with TGA after atrial switch and 6 (33%) patients with ccTGA. N-terminal pro-B-type natriuretic peptide (NT-pro-BNP) decreased significantly (950–358 ng/L, p<0.001). Echocardiographic systemic RV fractional area change and global longitudinal strain showed small improvements (19%–22%, p<0.001 and −11% to −13%, p=0.014, respectively). The 6 min walking distance improved significantly from an average of 564 to 600 m (p=0.011). The QOL domains of cognitive function, sleep and vitality improved (p=0.015, p=0.007 and p=0.037, respectively).

**Conclusions:**

We describe the first patient cohort with systemic RV failure treated with sacubitril/valsartan. Treatment appears feasible with improvements in NT-pro-BNP and echocardiographic function. Our positive results show the potential of sacubitril/valsartan for this patient population.

## Introduction

Patients with transposition of the great arteries (TGA) who underwent an atrial switch procedure according to Mustard or Senning constitute an important group within the clinical setting of adult patients with congenital heart disease. Together with patients with congenitally corrected transposition of the great arteries (ccTGA), they represent a cohort of patients with a systemic right ventricle (RV)—a situation in which the morphological RV is in subaortic position and sustains the systemic circulation. Although mid-term survival in this group is good, failure of the systemic RV is, in the long term, inevitable.^1 2^ Furthermore, tricuspid valve regurgitation (TR), conduction abnormalities, arrhythmias and myocardial perfusion defects are frequently encountered and complicate the course of heart failure in these patients.^1 2^

Compared with treatment of systolic heart failure in patients with a systemic left ventricle (LV), pharmacological options in patients with a systemic RV are currently less well defined. Data regarding effectiveness of drug therapy in the latter group are scarce and extrapolation from the guidelines and recommendations on LV failure is inappropriate due to specific anatomic and haemodynamic characteristics of the systemic RV. Although beta-blockers provide beneficial effects at higher doses, this may result in clinically important bradycardia in the atrioventricular conduction abnormalities prone patients with ccTGA.^3^ Beta-blockers and ACE inhibitors (ACEi)/angiotensin II-receptor blockers (ARB) are prescribed based on carefully optimistic results from several, mostly small, trials and retrospective studies.^4–9^

The largest trial regarding medical treatment of the failing systemic RV investigated the effects of valsartan in 88 patients with congenitally or by an atrial switch corrected TGA.^9^ In symptomatic patients in the placebo group, the RV ejection fraction deteriorated significantly, whereas in the valsartan group, the ejection fraction remained stable over 3 years of follow-up. Longer follow-up of this cohort showed fewer events in symptomatic patients in the valsartan group,^10^ suggesting that adequate medical therapy can impact the long-term outcomes in patients with systemic RV failure.

The treatment of symptomatic systolic LV heart failure has improved since the introduction of the combination drug sacubitril/valsartan, resulting in positive effects in clinical outcomes as well as in beneficial structural and functional cardiac changes.^11^ The combination of sacubitril and valsartan was superior to enalapril in reducing the risk of death and hospitalisation for systolic heart failure of patients with acquired heart disease, all with a systemic LV.^11^

Neurohormonal activation has been shown to be related to symptom severity and systemic ventricular dysfunction in patients with congenital heart disease.^12^

In patients with a systemic RV, the N-terminal pro-B-type natriuretic peptide (NT-pro-BNP) levels have a predictive value in clinical end points, including mortality.^13 14^ Furthermore, brain natriuretic peptide (BNP) has been shown to correlate with systemic RV dysfunction.^15^ Sacubitril is a neprilysin inhibitor. Neprilysin, a neutral endopeptidase, degrades several endogenous vasoactive peptides, including natriuretic peptides and bradykinin, but not NT-pro-BNP. Inhibition of neprilysin increases the levels of these substances, countering the neurohormonal overactivation that leads to vasoconstriction, sodium retention and maladaptive remodelling in heart failure. Although BNP and NT-pro-BNP have both been proven to be useful biomarkers, the levels of BNP often fluctuate during heart failure therapy (attributable to inhibition of neprilysin), whereas decrease in NT-pro-BNP levels has been correlated with improvements in heart failure condition. This could lead to clinical confusion and the use of NT-pro-BNP has been preferred and recommended.^11 16–18^

Sacubitril/valsartan treatment is currently indicated in all symptomatic patients with heart failure with an ejection fraction ≤35% already treated with a β-blocker and an ACEi or ARB.^19^ However, no studies are yet available evaluating the effects of sacubitril/valsartan on heart failure in the systemic RV population. The current study aims to investigate the feasibility and effects of sacubitril/valsartan treatment in this group of patients in a single-centre cohort.

## Methods

### Design and inclusion/exclusion criteria

In this single-centre (Leiden University Medical Center) cohort study, data of all consecutive adult patients with a failing systemic RV in a biventricular circulation treated with sacubitril/valsartan are reported. In 2018, all adult patients with systemic RV heart failure (n=67) were screened for eligibility for treatment with sacubitril/valsartan. Those who had an (estimated) systemic RV ejection fraction of ≤35% (defined as a moderately to severely reduced systemic RV function on echocardiography and/or MRI) and remained symptomatic despite treatment with highest tolerated doses of a β-blocker and an ACEi or ARB for a period of at least 3 months were advised to start treatment with sacubitril/valsartan.^11^ Symptoms were assessed based on the history and complaints as reported at the outpatient clinic (including the patients’ performance during work and sport activities) and/or heart failure related admissions or ambulant medication adjustments (increasing diuretic dose to remain euvolemic). Patients with a ventricular assist device (VAD or awaiting the implantation of a VAD) or severe renal function impairment (estimated glomerular filtration rate (eGFR) <30 mL/min/1.73 m2) were excluded.

### Treatment and follow-up

Before the initiation of sacubitril/valsartan treatment, the following investigations were performed as part of routine clinical care: echocardiography, bicycle exercise test with VO_2_max, a 6 min walking test (MWT), laboratory investigation including haemoglobin levels, kidney function, electrolytes and NT-pro-BNP and a quality of life (QOL) assessment. QOL was assessed with the Netherlands Organisation for applied scientific research/Academic Hospital Leiden adult Quality Of Life questionnaire (TAAQOL), which has been used previously to assess health-related QOL in adults with congenital heart disease.^20^ It includes 45 questions and evaluates 12 different components of health-related QOL.

**Figure 1:**
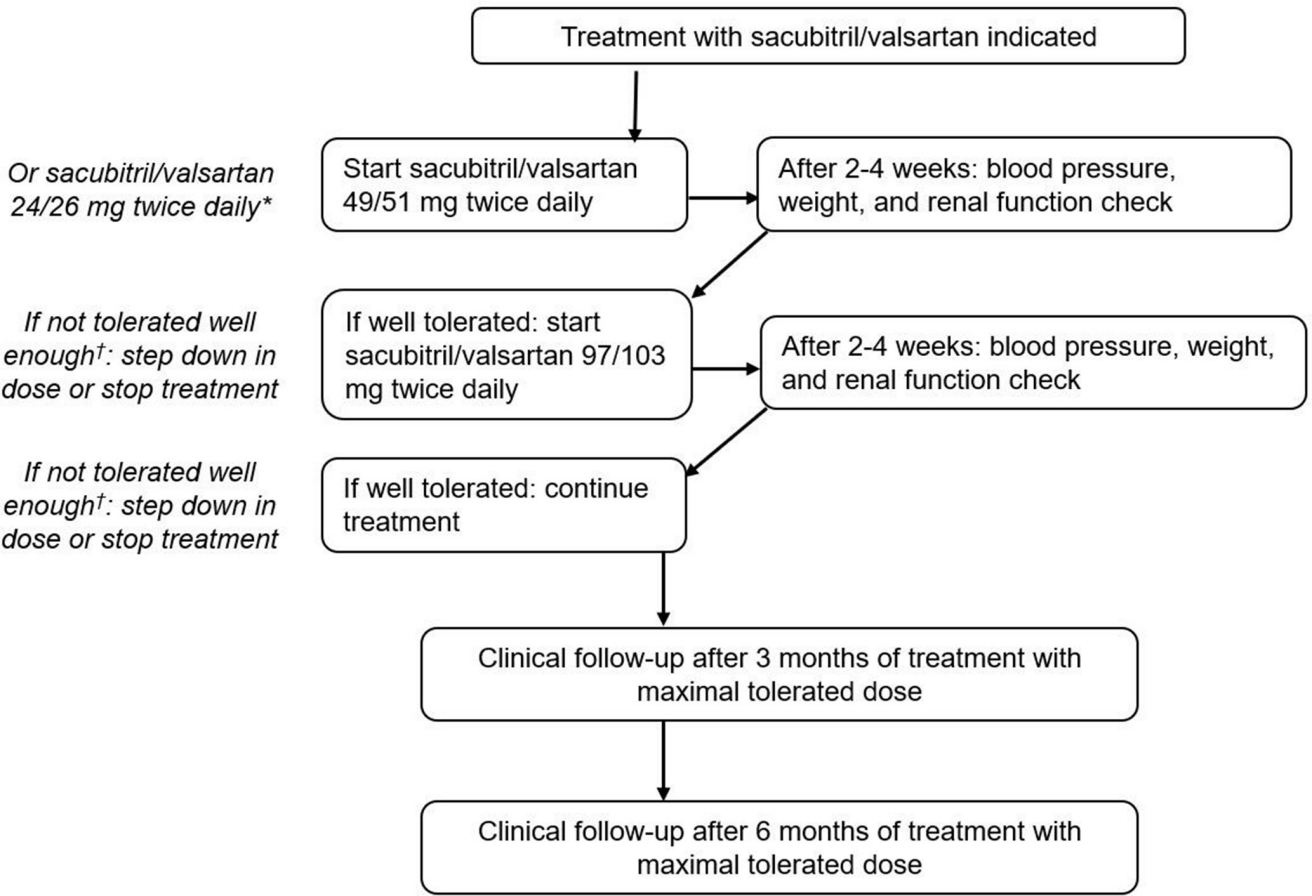
Treatment and follow-up protocol. *Depending on previous dose of ACEi/ARB. †If potassium >5.5 mmol/L, increase in creatinine >310 μmol/L (or eGFR <20 mL/min/1.73 m^2^), hypotension or signs of decompensation. ACEi, ACE inhibitors; ARB, angiotensin II-receptor blockers.

Figure 1 shows the treatment protocol. Depending on the previously used dose of ACEi or ARB, the starting dose of sacubitril/valsartan was chosen: if patients were using at least 80 mg of valsartan two times a day or an equivalent dose (perindopril 4 mg one time a day, lisinopril 20 mg one time a day, enalapril 20 mg one time a day, losartan 100 mg one time a day, irbesartan 300 mg one time a day, telmisartan 20 mg one time a day or candesartan 32 mg one time a day), the starting dose of sacubitril/valsartan was 49/51 mg two times a day. If the previous dose of ACEi/ARB was less than (the equivalent of) valsartan 80 mg two times a day, the starting dose of sacubitril/valsartan was 24/26 mg two times a day.^19^ Patients were instructed to wait 36 hours after taking the last dose of ACEi prior to initiating treatment with sacubitril/valsartan to reduce the risk of angioedema.^11 19^

After 2–4 weeks of treatment with the starting dose, blood pressure, weight, kidney function and complaints were evaluated. If the medication was well tolerated, the dose of sacubitril/valsartan was increased in a stepwise fashion until the highest tolerated dose was reached. Potassium increase to >5.5 mmol/L and/or an increase in creatinine >221μmol/L (or eGFR <30 mL/min/1.73m^2^) was followed by a step down in the dose and follow-up after 2–4 weeks. In the case of potassium increase >6 mmol/L and/or an increase in creatinine >310 μmol/L (or eGFR <20 mL/min/1.73 m^2^), the medication was stopped. Symptomatic hypotension and/or signs of decompensation were also followed by a stepdown or termination of treatment.

After 3 months of treatment with the optimal tolerated dose, blood pressure, weight, adverse events and laboratory investigations (including haemoglobin levels, kidney function, electrolytes and NT-pro-BNP) were repeated. After 6 months, echocardiography, bicycle exercise test with VO_2_max, a 6-MWT and laboratory investigations were re-evaluated, combined with physical examination and the TAAQOL questionnaire.

The serial echocardiograms were performed with commercially available ultrasound systems and were analysed offline in EchoPAC, GE Medical Systems. The echocardiographic parameters were assessed and measured offline by two cardiologists with expertise in congenital imaging blinded to the study (all echocardiograms were performed as standard of care and clinical follow-up at our centre).

### Ethics statement

All tests and procedures performed involving human participants were in accordance with the ethical standards of the institutional and/or national research committee and with the 2013 Helsinki declaration or comparable ethical standards. Appropriate local scientific board approval was obtained and the need for written informed consent was waived by the institutional medical ethical board. All patients provided consent for registration of their data and publication.

### Patient and public involvement

Patients were informed about the research process and the background knowledge at initiation of treatment. The research questions and outcome measures were developed in consensus between the researchers and the treating cardiologists of this patient group (based on their extensive clinical experience). All the tests and procedures described were part of optimal patient care. The study results will be disseminated through national and local patient information websites.

### Statistical analysis

All statistical analyses were performed in IBM SPSS V.23. Normally distributed continuous data are displayed as mean±SD and non-normally distributed continuous data are displayed as median (IQR). Proportions are displayed as numbers (percentages). For the comparison of values over time, paired samples t-tests or Wilcoxon rank tests were used as appropriate. A value of p<0.05 was considered to be statistically significant.

## Results

### Baseline clinical characteristics and sacubitril/valsartan initiation

Between January and August 2019, 20 consecutive patients with systemic RV failure who fulfilled the inclusion criteria initiated treatment with sacubitril/valsartan (online supplemental table 1). Mean age was 46±11 years, and 50% were women. In one patient (patient 7), sacubitril/valsartan treatment was discontinued due to uncontrollable thirst with subsequent ample fluid intake and admission with cardiac decompensation. Another patient who was in end-stage heart failure at baseline (patient 8) declined screening for a VAD and died of progressive cardiogenic shock despite initiating treatment with sacubitril/valsartan. The 18 remaining patients were further analysed. Twelve patients (67%) had TGA corrected with the Mustard or Senning atrial switch procedure, and six patients (33%) had ccTGA.

10.1136/heartjnl-2020-318074.supp1 Supplementary data

The target dose of 97/103 mg sacubitril/valsartan two times a day was reached in 12 (67%) patients. Four patients (22%) had a maximum tolerated dose of 49/51 mg two times a day and two patients (11%) had a maximum tolerated dose of 24/26 mg two times a day. The reason to not further increase the dosage was symptomatic hypotension in all cases. None of the patients developed clinically relevant hyperkalaemia or significant deterioration of the renal function.

### Follow-up after six months of treatment

#### Laboratory results

**Figure 2:**
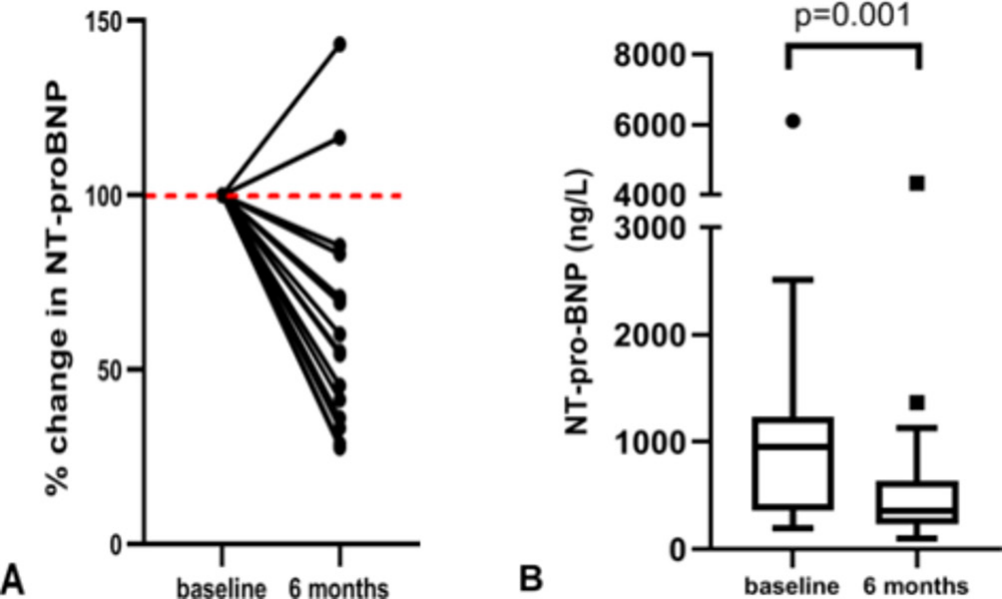
(A) Percentage of change in NT-pro-BNP at 6 months compared with the levels of individual patients at baseline. (B) Absolute NT-pro-BNP levels at baseline and 6 months, showing median (horizontal line) with IQR (box), lower and upper extreme (whiskers) and outliers (values represented with ● and ■). NT-pro-BNP, N-terminal pro-B-type natriuretic peptide.

There was a significant decrease in NT-pro-BNP after 6 months of sacubitril/valsartan use (median 950–358 ng/L, p<0.001, table 1). Relative reduction of NT-pro-BNP per patient is (percentage from baseline levels) shown in figure 2. Overall, the median reduction in NT-pro-BNP was 45% (IQR 26–60) of the value at treatment initiation. In two patients (patient 15 and 19), there was an increase in NT-pro-BNP.

**Table 1:** Changes in the laboratory values 6 months after initiation of sacubitril/valsartan treatment, n=18

Laboratory values	Mean±SD or median (IQR) Baseline	Mean±SD or median (IQR) 6 months	P value
Hb (mmol/L)	8.7±0.9	8.9±0.9	0.083
Ht (L/L)	0.42±0.04	0.43±0.04	0.004*
MCV (fL)	89±5	91±3	0.006*
MCH (fmol)	1.84±0.12	1.86±0.07	0.251
RDW (%)	13.0±1.2	12.9±1.1	0.252
Sodium (mmol/L)	140±2	141±2	0.182
Potassium (mmol/L)	4.3±0.4	4.5±0.3	0.011*
Creatinine (μmol/L)	86±18	89±14	0.095
eGFR (mL/min/1.73 m^2^)	85±20	80±21	0.087
BUN (mmol/L)	6.5 (5.5–6.8)	6.3 (4.9–7.5)	0.649
ASAT (U/L)	31±13	29±13	0.162
ALAT (U/L)	30±11	26±12	0.013*
Gamma GT (U/L)	39 (26–82)	43 (27–62)	0.767
Total bilirubin (μmol/L)	11.5 (7.8–18.3)	10 (8.0–17.0)	0.550
CK (U/L)	93±40	105±58	0.328
Troponin T (ng/L)	11.0 (5.3–16.8)	7.5 (6.0–11.3)	0.109
NT-pro-BNP (ng/L)	950 (364–1235)	358 (233–639)	<0.001*

*Statistically significant.

ALAT, alanine transaminase; ASAT, aspartase aminotransferase; BUN, blood urea nitrogen; Gamma GT, gamma glutamyltransferase; Hb, haemoglobin; Ht, haematocrit; MCH, mean corpuscular haemoglobin; MCV, mean corpuscular volume; NT-pro-BNP, N-terminal pro–B-type natriuretic peptide; RDW, red blood cell distribution width.;

There was a significant increase in both haematocrit and mean corpuscular volume (MCV), and a significant decrease in alanine transaminase (ALAT) levels. There was a statistically significant, but clinically irrelevant, increase in potassium and the eGFR did not change significantly (table 1).

### Echocardiography

There was an improvement in echocardiographic systemic RV function as accessed by the fractional area change (p<0.001) and in echocardiographic RV global longitudinal strain values (p=0.014) (table 2). The global assessment of RV function using eye balling technique, the RV end diastolic diameter and severity of tricuspid regurgitation did not change significantly following 6 months of treatment. The function of the subpulmonary LV remained stable.

**Table 2:** Changes in physical examination, echocardiography and functional status 6 months after initiation of sacubitril/valsartan treatment, n=18

Variable	Mean±SD Baseline	Mean±SD 6 months	P value
*General*			
NYHA class (n, %)			0.112
II	13 (72%)	15 (83%)	
III–IV	5 (28%)	3 (17%)	
Systolic blood pressure (mm Hg)	106±10	106±14	0.960
Weight (kg)	80±18	79±18	0.187
6 min walking distance (m)	564±104	600±72	0.011*
Echocardiography:			
Global RV function, (n, %)			0.157
Mildly reduced	4 (22%)	5 (28%)	
Moderately reduced	10 (56%)	10 (56%)	
Severely reduced	4 (22%)	3 (16%)	
TAPSE (mm)	12±2	11±2	0.211
RV FAC (%)	19±7	22±7	<0.001*
RV GLS (%)	−11±3	−13±2	0.014*
RVEDD (mm)	59±9	58±8	0.067
Tricuspid valve regurgitation (n, %)			1.000
Grade I–II	15 (88%)	15 (88%)	
Grade III–IV	2 (12%)	2 (12%)	
LV GLS (%)	−16±4	−18±5	0.110
MAPSE (mm)	18±5	18±3	0.663
*Exercise testing*			
Exercise capacity (W)	129±50	132±47	0.402
Exercise capacity (%)	79±17	81±17	0.575
VO_2_max (ml/min/kg)	18±5	18±4	0.886
% of predicted VO_2_max achieved	59±15	58±13	0.746
% of predicted heart rate achieved	77±13	78±14	0.717
Heart rate reserve (bpm)	66±24	65±26	0.795
RER	1.20±0.09	1.17±0.07	0.367

*Statistically significant.

bpm, beats per minute; FAC, fractional area change; GLS, global longitudinal strain; LV, (subpulmonary) left ventricle; MAPSE, mitral annular plane systolic excursion; NYHA, New York Heart Association functional classification; RER, respiratory exchange ratio; RV, (systemic) right ventricle; RVEDD, (systemic) right ventricular end diastolic diameter (basal measurement); TAPSE, tricuspid annular plane systolic excursion.

### Clinical characteristics

The 6 min walking distance slightly increased from a mean of 564–600 m (p=0.011). The NYHA functional class, blood pressure, weight and maximal exercise capacity as assessed with exercise testing remained stable and none of the patients showed clinical deterioration during the study period (table 2).

### Quality of life

Sixteen patients completed the TAAQOL questionnaire at both time points (response rate 89%). The results are shown in table 3. Higher scores (maximum 100) indicate higher QOL. After 6 months of treatment with sacubitril/valsartan, QOL regarding cognitive function, sleep and vitality domains improved significantly (p=0.015, p=0.007 and p=0.037, respectively, table 3). In the other domains, there were no significant changes.

**Table 3:** Quality of life as assessed with the TAAQOL questionnaire, n=16

Scales	Mean±SD or median (IQR) Baseline	Mean±SD or median (IQR) 6 months	P value
Cognitive function	72 (38–98)	91 (70–100)	0.015*
Sleep	50 (38–86)	88 (36–100)	0.007*
Pain	75 (41–100)	78 (63–100)	0.152
Social functioning	97 (88–100)	100 (78–100)	1.000
Daily activities	72 (34–98)	84 (64–98)	0.395
Sexuality	100 (25–100)	100 (47–100)	0.344
Vitality	49±32	64±27	0.037*
Positive emotions	68±28	71±23	0.657
Depressive emotions	75±25	75±20	0.942
Aggressive emotions	100 (81–100)	100 (81–100)	0.673

## Discussion

In this study, the tolerability and the effects of sacubitril/valsartan treatment in a single-centre cohort of adult patients with systemic RV failure were evaluated. Six months of treatment resulted in (1) a significant reduction in NT-pro-BNP levels (2) a subtle improvement in systemic RV function as assessed by echocardiography and (3) improvement in the 6 min walking distance and health-related QOL, without high rates of treatment discontinuation, symptomatic hypotension, hyperkalaemia or renal function decline.

This is the first cohort of patients with systemic RV heart failure treated with sacubitril/valsartan. As opposed to the recent work of Maurer *et al*, our findings show that this specific group of patients can indeed benefit from treatment with sacubitril/valsartan.^21^ Previous studies with smaller cohorts or populations mixed with other types of congenital heart defects show neutral or tentatively positive results but do not perform formal statistics or do not concern patients with systemic RV specifically.^21 22^ Our findings provide new insight into the pharmacological possibilities of heart failure treatment in the patients with systemic RV and justify assessment in larger prospective cohorts.

Secondary analysis of the cohort described in the landmark Prospective Comparison of ARNI with ACEI to Determine Impact on Global Mortality and Morbidity in Heart Failure (PARADIGM-HF) trial showed a median decrease of NT-pro-BNP of 28% after the first 8–10 weeks of treatment.^16^ Furthermore, the proportion of patients in which the treatment was limited by symptomatic hypotension, hyperkalaemia or a decline in renal function in the PARADIGM- HF trial was low.^11^ In the current cohort, the median decrease in NT-pro-BNP was 45% after 6 months of treatment. There were no cases in which titration was halted or treatment had to be discontinued due to hyperkalaemia or decline in renal function, perhaps reflecting on the usually preserved subpulmonary function of the anatomic LV and preserved renal function. The observed rise of haematocrit, MCV and decrease in ALAT levels may be attributed to a net excretion of excess volume and subsequent relief of the hemodynamically congested liver. In a third of the patients, the maximum dose of 97/103 mg two times a day was not reached due to symptomatic hypotension, but lower doses were well tolerated. Similar proportions of patients reaching the maximum dose are described in other retrospective cohorts in patients with non-congenital heart disease and the maximally tolerated dose remains a matter of individualised patient care.^23^

Identification of patients in the early stages of deterioration of systemic RV function remains a challenge as patients with complex congenital heart disease are typically used to living with limitations in their exercise tolerance and detection of subtle changes using routine echocardiography is technically challenging. Despite this, finding the optimal window for optimisation of medical therapy to stimulate reverse myocardial remodelling and improve the long-term outcome is crucial. BNP and NT-pro-BNP levels may provide a useful clinical tool in identifying and managing adult patients with congenital heart disease.^24^ The correlation of NT-pro-BNP and clinical and echocardiographic parameters in our study is illustrative of this. A recent study evaluating medication use in adults after atrial switch for TGA showed that only the symptomatic patients with systemic RV benefited from the use of heart failure medication, suggesting that adequate patient selection is key and discouraging prophylactic use of heart failure medication in this patient group.^25^

A recent study of sacubitril/valsartan in an animal model of RV pressure overload showed that sacubitril/valsartan prevented maladaptive RV remodelling by diminishing the effective RV pressure increase, hypertrophy, collagen and myofibre reorientation and amelioration of tissue stiffening. This provides some insight into the potential mechanism of action of sacubitril/valsartan in the failing (systemic) RV.^26^

Of interest is the discrepancy between the only slightly reduced distance attained during the 6-MWT (564 m), which improved after 6 months of treatment with sacubitril/valsartan and the unaffected, poor performance as assessed by exercise testing and VO_2_max (VO_2_max 18 mL/min/kg, 59% of predicted) in our study population. Patients with preserved systemic RV function are known to have significantly lower peak and anaerobic threshold oxygen uptake compared with age matched controls. In addition, impaired systemic RV function further reduces the peak oxygen uptake.^27^ Perhaps the limited contractile reserve and poor tolerance of pressure overload of the systemic RV can explain the poor performance during peak exercise training such as seen during the exercise testing. Although the 6-MWT is best reflective of low intensity exercise capacity, most daily activities are of low intensity and therefor the observed improvement in 6-MWT performance is promising.

As compared with the general population, adults with congenital heart disease have a worse self-perceived health-related QOL. This may be even worse in patients with complex congenital defects.^20^ Secondary analysis of the PARADIGM-HF trial showed improvement in QOL in both physical and mental domains.^28^ In the current cohort, improvements were seen in the domains of cognitive function, sleep and vitality after 6 months of treatment with sacubitril/valsartan, suggesting that, although this was an open-label study, the self-perceived QOL can indeed be improved with medical intervention.

### Study limitations

This study is limited by its single-arm, non-blinded design and the relatively small study population, reflective of the rarity of the condition. The findings should be interpreted taking into account the open-label nature of the study. Therefore, the results should be confirmed in a larger, preferably randomised, double-blinded and placebo-controlled trial.

## Conclusion

This is the first cohort of patients with systemic RV heart failure treated with sacubitril/valsartan. This appears to be well tolerated and leads to improvements in NT-pro-BNP and echocardiographic function. The positive results show the potential of sacubitril/valsartan in the treatment of this patient population.

## Clinical perspectives

With the current survival rates of 82% at 40 years after atrial switch operation for TGA and 84% survival at the age of 40 in patients with ccTGA, the burden of systemic RV heart failure in this young population will increase over the next decades.^29^ Heart failure treatment in this patient group remains a challenge. Optimisation of pharmacological treatment, aggressive treatment of arrhythmias, the search of adequate pacing modalities including resynchronisation therapy and timely surgical treatment of TR all play an important role in halting the progression of systemic RV heart failure. The specific anatomical and physiological characteristics, together with extensive surgical history and scarce numbers of donor hearts often makes this group unsuitable for cardiac transplantation. When confronted with advanced heart failure, VAD implantation as destination therapy shows promising results in patient with systemic RV failure.^30^ The present study demonstrates that sacubitril/valsartan results in improved RV function, exercise capacity and QOL in symptomatic patients with systemic RV.

**Key messages**

**What is already known on this subject?**

- Patients with systemic right ventricular (RV) heart failure are frequently encountered in congenital heart disease and represent a distinct anatomical, pathophysiological and clinical entity. Pharmacological options for patients with a failing systemic RV in the context of transposition of the great arteries (TGA) after atrial switch or congenitally corrected TGA are not well defined and the feasibility and effects of sacubitril/valsartan in the treatment of these patients have not yet been evaluated.

**What might this study add?**

- We describe the first patient cohort with systemic RV failure treated with sacubitril/valsartan. Treatment appears feasible and results in improvements in N-terminal pro-B-type natriuretic peptide, echocardiographic function, walking distance and self-assessed quality of life.

**How might this impact on clinical practice?**

- The positive results show the potential of sacubitril/valsartan in the treatment of this heart-failure prone and often young patient population.

## Data Availability

All data relevant to the study are included in the article or uploaded as supplementary information. Additional data (statistical analysis plans) are available on reasonable request.

## References

[1] Filippov AA , Del Nido PJ , Vasilyev NV . Management of systemic right ventricular failure in patients with congenitally corrected transposition of the great arteries. Circulation 2016;134:1293–302. 10.1161/CIRCULATIONAHA.116.022106 27777298

[2] Vejlstrup N , Sørensen K , Mattsson E , et al . Long-term outcome of Mustard/Senning correction for transposition of the great arteries in Sweden and Denmark. Circulation 2015;132:633–8. 10.1161/CIRCULATIONAHA.114.010770 26185211

[3] Baumgartner H , Bonhoeffer P , De Groot NMS , et al . ESC guidelines for the management of grown-up congenital heart disease (new version 2010). Eur Heart J 2010;31:2915–57. 10.1093/eurheartj/ehq249 20801927

[4] Bouallal R , Godart F , Francart C , et al . Interest of β-blockers in patients with right ventricular systemic dysfunction. Cardiol Young 2010;20:615–9. 10.1017/S1047951110000764 20519056

[5] Dore A , Houde C , Chan K-L , et al . Angiotensin receptor blockade and exercise capacity in adults with systemic right ventricles: a multicenter, randomized, placebo-controlled clinical trial. Circulation 2005;112:2411–6. 10.1161/CIRCULATIONAHA.105.543470 16216961

[6] Doughan ARK , McConnell ME , Book WM . Effect of beta blockers (carvedilol or metoprolol XL) in patients with transposition of great arteries and dysfunction of the systemic right ventricle. Am J Cardiol 2007;99:704–6. 10.1016/j.amjcard.2006.10.025 17317376

[7] Lester SJ , McElhinney DB , Viloria E , et al . Effects of losartan in patients with a systemically functioning morphologic right ventricle after atrial repair of transposition of the great arteries. Am J Cardiol 2001;88:1314–6. 10.1016/S0002-9149(01)02098-7 11728365

[8] Tutarel O , Meyer GP , Bertram H , et al . Safety and efficiency of chronic ACE inhibition in symptomatic heart failure patients with a systemic right ventricle. Int J Cardiol 2012;154:14–16. 10.1016/j.ijcard.2010.08.068 20843567

[9] van der Bom T , Winter MM , Bouma BJ , et al . Effect of valsartan on systemic right ventricular function: a double-blind, randomized, placebo-controlled pilot trial. Circulation 2013;127:322–30. 10.1161/CIRCULATIONAHA.112.135392 23247302

[10] van Dissel AC , Winter MM , van der Bom T , et al . Long-term clinical outcomes of valsartan in patients with a systemic right ventricle: follow-up of a multicenter randomized controlled trial. Int J Cardiol 2019;278:84–7. 10.1016/j.ijcard.2018.11.027 30449692

[11] McMurray JJV , Packer M , Desai AS , et al . Angiotensin-neprilysin inhibition versus enalapril in heart failure. N Engl J Med 2014;371:993–1004. 10.1056/NEJMoa1409077 25176015

[12] Bolger AP , Sharma R , Li W , et al . Neurohormonal activation and the chronic heart failure syndrome in adults with congenital heart disease. Circulation 2002;106:92–9. 10.1161/01.CIR.0000020009.30736.3F 12093776

[13] Popelová JR , Tomková M , Tomek J . NT-proBNP predicts mortality in adults with transposition of the great arteries late after mustard or Senning correction. Congenit Heart Dis 2017;12:448–57. 10.1111/chd.12466 28419713

[14] Westhoff-Bleck M , Podewski E , Tutarel O , et al . Prognostic value of NT-proBNP in patients with systemic morphological right ventricles: a single-centre experience. Int J Cardiol 2013;169:433–8. 10.1016/j.ijcard.2013.10.014 24169536

[15] Chow P-C , Cheung EW-Y , Chong C-Y , et al . Brain natriuretic peptide as a biomarker of systemic right ventricular function in patients with transposition of great arteries after atrial switch operation. Int J Cardiol 2008;127:192–7. 10.1016/j.ijcard.2007.06.004 17643533

[16] Myhre PL , Vaduganathan M , Claggett B , et al . B-Type Natriuretic Peptide During Treatment With Sacubitril/Valsartan: The PARADIGM-HF Trial. J Am Coll Cardiol 2019;73:1264–72. 10.1016/j.jacc.2019.01.018 30846338 PMC7955687

[17] Yancy CW , Januzzi JL , Allen LA , et al . 2017 ACC Expert Consensus Decision Pathway for Optimization of Heart Failure Treatment: Answers to 10 Pivotal Issues About Heart Failure With Reduced Ejection Fraction: A Report of the American College of Cardiology Task Force on Expert Consensus Decision Pathways. J Am Coll Cardiol 2018;71:201–30. 10.1016/j.jacc.2017.11.025 29277252

[18] Langenickel TH , Dole WP . Angiotensin receptor-neprilysin inhibition with LCZ696: a novel approach for the treatment of heart failure. Drug Discov Today 2012;9:e131–9. 10.1016/j.ddstr.2013.11.002

[19] Ponikowski P , Voors AA , Anker SD , et al . 2016 ESC Guidelines for the diagnosis and treatment of acute and chronic heart failure: The Task Force for the diagnosis and treatment of acute and chronic heart failure of the European Society of Cardiology (ESC)Developed with the special contribution of the Heart Failure Association (HFA) of the ESC. Eur Heart J 2016;37:2129–200. 10.1093/eurheartj/ehw128 27206819

[20] Fteropoulli T , Stygall J , Cullen S , et al . Quality of life of adult congenital heart disease patients: a systematic review of the literature. Cardiol Young 2013;23:473–85. 10.1017/S1047951112002351 23388149

[21] Maurer SJ , Pujol Salvador C , Schiele S , et al . Sacubitril/valsartan for heart failure in adults with complex congenital heart disease. Int J Cardiol 2020;300:137–40. 10.1016/j.ijcard.2019.06.031 31242968

[22] Lluri G , Lin J , Reardon L , et al . Early experience with Sacubitril/Valsartan in adult patients with congenital heart disease. World J Pediatr Congenit Heart Surg 2019;10:292–5. 10.1177/2150135119825599 31084317

[23] Du AX , Westerhout CM , McAlister FA , et al . Titration and tolerability of Sacubitril/Valsartan for patients with heart failure in clinical practice. J Cardiovasc Pharmacol 2019;73:149–54. 10.1097/FJC.0000000000000643 30540684

[24] Baggen VJM , Baart SJ , van den Bosch AE , et al . Prognostic value of serial N-terminal pro-B-type natriuretic peptide measurements in adults with congenital heart disease. J Am Heart Assoc 2018;7:e008349. 10.1161/JAHA.117.008349 29581225 PMC5907602

[25] Woudstra OI , Kuijpers JM , Jongbloed MRM , et al . Medication in adults after atrial switch for transposition of the great arteries: clinical practice and recommendations. Eur Heart J Cardiovasc Pharmacother 2020. 10.1093/ehjcvp/pvaa111. [Epub ahead of print: 25 Sep 2020]. PMC8728040 32976560

[26] Sharifi Kia D , Benza E , Bachman TN , et al . Angiotensin Receptor-Neprilysin inhibition attenuates right ventricular remodeling in pulmonary hypertension. J Am Heart Assoc 2020;9:e015708. 10.1161/JAHA.119.015708 32552157 PMC7670537

[27] Rog B , Salapa K , Okolska M , et al . Clinical evaluation of exercise capacity in adults with systemic right ventricle. Tex Heart Inst J 2019;46:14–20. 10.14503/THIJ-17-6408 30833832 PMC6379015

[28] Lewis EF , Claggett BL , McMurray JJV , et al . Health-related quality of life outcomes in PARADIGM-HF. Circ Heart Fail 2017;10:e003430. 10.1161/CIRCHEARTFAILURE.116.003430 28784687

[29] Couperus LE , Vliegen HW , Zandstra TE , et al . Long-term outcome after atrial correction for transposition of the great arteries. Heart 2019;105:790–6. 10.1136/heartjnl-2018-313647 30415204

[30] Zandstra TE , Palmen M , Hazekamp MG , et al . Ventricular assist device implantation in patients with a failing systemic right ventricle: a call to expand current practice. Neth Heart J 2019;27:590–3. 10.1007/s12471-019-01314-y 31420818 PMC6890896

