# Supplementary material for: Sacubitril/valsartan in the treatment of systemic right ventricular failure
Source: Heart. 2021 Jan 15;107(21):1725–30. doi: 10.1136/heartjnl-2020-318074 (PMC8522462; doi:10.1136/heartjnl-2020-318074)
Supplement: Supplementary data [file heartjnl-2020-318074supp001.pdf]

BMJ Publishing Group Limited (BMJ) disclaims all liability and responsibility arising from any reliance placed on this supplemental material which has been supplied by the author(s)

Supplementary material
Table 1

Zandstra TE, *et al. Heart* 2021; 107:1725–1730. doi: 10.1136/heartjnl-2020-318074

PATIENT	AGE (years)	SEX	ANATOMY	CONGENITAL DEFECTS	CONCOMITANT SURGERY OR RE-OPERATIONS	CARDIAC MEDICATION AT BASELINE	DEVICE	MAXIMAL TOLERATED DOSE SACUBITRIL/VALSARTAN	EVENTS
**1**	52	F	ccTGA	ccTGA, VSD	VSD closure	Phenprocoumon, hydrochlorothiazide, losartan, metoprolol, isosorbide mononitrate		49/51	
**2**	21	M	Senning	TGA, CoA, intramural coronary artery		Acenocoumarol, enalapril, spironolacton, metoprolol	CRT-D	97/103	
**3**	42	F	Senning	TGA	TVP	Phenprocoumon, bumetanide, spironolactone, atenolol	DDD-ICD	49/51	
**4**	57	F	Mustard	TGA, VSD, ASD, PS	TVP	Acenocoumarol, lisinopril, bumetanide, triamterene, amiodarone, digoxin	DDD-ICD	49/51	
**5**	47	M	Mustard	TGA		Phenprocoumon , valsartan, furosemide, spironolactone, sotalol	PM	97/103	
**6**	42	M	Senning	TGA		Irbesartan		97/103	
**7**	26	F	ccTGA	ccTGA, TI	TVR	Phenprocoumon, losartan, spironolactone	CRT-P	49/51	Stopped: Increased thirst stimulus, resulting in decompensation)
**8**	72	F	ccTGA	ccTGA		Phenprocoumon, perindopril/indapamide, spironolactone, furosemide,	PM	49/51	Died: refractory heart failure, started sacubitril/valsartan as a 'last resort'

Zandstra TE, *et al. Heart* 2021; 107:1725–1730. doi: 10.1136/heartjnl-2020-318074

						metoprolol, amiodarone			
**9**	36	M	ccTGA	Dextrocardia, ccTGA, PA, VSD	VSD closure, homograft PV, AVRepair	Perindopril		97/103	
**10**	44	M	Mustard	TGA		Aspirin, valsartan	CRT-D	97/103	
**11**	43	M	Senning	TGA, VSD		Apixaban, irbesartan, bisoprolol		97/103	
**12**	49	M	Mustard	TGA, VSD	VSD closure	Irbesartan, metoprolol, amiodarone	DDD-ICD	97/103	
**13**	45	F	Senning	TGA, VSD, PS	PV repair	Clopidogrel, irbesartan, furosemide, spironolactone, carvedilol		49/51	
**14**	46	M	Mustard	TGA		Phenprocoumon, irbesartan, bumetanide, metoprolol	DDD-ICD	97/103	
**15**	43	F	Senning	TGA, VSD, PS	VSD closure, PV Repair	Phenprocoumon, perindopril, bumetanide, spironolactone, metoprolol	PM	24/26	Multiple admissions for SVTs, and ablation procedures
**16**	49	F	Mustard	TGA		Perindopril, furosemide, bisoprolol	DDD-ICD	97/103	Admission for SVT with inappropriate ICD shock
**17**	45	F	ccTGA	ccTGA, PS		Enalapril		97/103	
**18**	56	F	ccTGA	Dextrocardia, ccTGA, VSD, PS	TVP	Phenprocoumon, candesartan, furosemide, spironolacton, digoxin	CRT-D	24/26	
**19**	57	M	ccTGA	Dextroversion, ccTGA, PS, ASD	closure ASD, PV repair	Acenocoumarol, irbesartan, hydrochlorothiazide, simvastatin		97/103	
**20**	45	M	ccTGA	ccTGA, VSD	TVR	Phenprocoumon, telmisartan		97/103	

Zandstra TE, *et al. Heart* 2021; 107:1725–1730. doi: 10.1136/heartjnl-2020-318074
